# Current News

**Published:** 1900-07

**Authors:** 


### Current News.

### INTERNATIONAL DENTAL CONGRESS.

A Reception Committee, composed of the following-named gentlemen, has been appointed by the management of the international Dental Congress at Paris to look after the welfare of residents of the United States attending the Dental Congress: Dr. du Bouchet, *President,* 8, Boulevard des Capucines; Dr. Roussell, *Secretary,* 74, Boulevard Haussmann; Drs. Barrett, Bogue, Crane, Daboll, I. B. Davenport, W. Davenport, Fay, Gries, Hotz, Lie, Levett, Mung, Georges Ryan, Jean Ryan, J. H. Spaulding, and Silva.

### MISSOURI STATE DENTAL ASSOCIATION.

The thirty-sixth annual meeting of the Missouri State Dental Association will be held at Louisiana, Mo., July 10, 11, 12, and 13, 1900. A cordial invitation is extended to all reputable dentists to be present and participate in the proceedings, and become members of the Association. All railroads in the State have granted a one-and-one-third fare on the certificate plan. The Palmer House hotel rates are $1.50 and $2.00 per day.

*Essays and Discussions.*—Hon. Champ Clark, "Address of Welcome;" Dr. W. L. Reed, Mexico, "President's Address;" Dr. W. H. DeFord, Cedar Rapids, Iowa, "Necrosis, involving the Alveolar Process, Superior Maxillary, Nasal and Palatal Bones, resulting from Maltreatment of an Alveolar Abscess;" Dr. J. B. Chaffee, "Things I have noticed and other Things;" Dr. Ira B. Crissman, Chicago, "Relation of Dental Colleges to the Profession and the General Public;" Dr. Harry M. Hill, St. Louis, "The Material, Porcelain;" Dr. H. E. Zorn, De Soto, "Finished Dentistry;" Dr. M. D. Hamisfar, Warrensburg, "Bacteriology and Pathology: Their Relation;" Dr. John G. Harper, St. Louis, "Incidents in Practice;" Dr. H. H. Sullivan, Kansas City, "Open-Faced Gold Crowns;" Dr. R. C. Brophy, Chicago, "The Universal Tooth;" Dr. Charles L. Van Fossen, Kansas City, Subject to be announced; Dr. C. D. Lukens, St. Louis, Lantern Lecture on "Orthodontia;" Dr. D. J. McMillen, Kansas City, "Preparation of Cavities and Filling with Non-Cohesive and Cohesive Gold-Foil" (with illustrations); Dr. J. T. Fry, Moberly, Mo., "Suggestions to the Young Practitioner;" Dr. D. F. Orr, Liberty, "Questions on Methods and Practice."

The clinics, beginning the second day at nine A.M., cover nearly all the operations in dentistry.

L. THORPE,
*Corresponding Secretary.*

ST. LOUIS.

### NEW YORK STATE DENTAL SOCIETY.

AT the annual meeting of the New York State Dental Society, held at Albany, May 9 and 10, 1900, the following officers were elected for the coming year: President, Dr. John I. Hart, New York; Vice-President, Dr. R. H. Hofheinz, Rochester; Secretary, Dr. W. A. White, Phelps; Treasurer, Dr. C. W. Stainton, Buffalo; Correspondent, Dr. H. D. Hatch, New York.

W. A. WHITE,
*Secretary.*

PHELPS, N. Y.

### IOWA STATE DENTAL SOCIETY.

AT the thirty-eighth annual meeting of the Iowa State Dental Society, held in Dubuque, May 1 to 3, the following officers were elected for the ensuing year: President, T. A. Gormly, Mt. Vernon; Vice-President, E. D. Brower, Le Mars; Secretary, I. C. Brownlie, Ames; Treasurer, W. R. Clack, Clear Lake.

I. C. BROWNLIE,
*Secretary.*

### REMOVAL.

DR. WILLIAM BALDWIN KEYES, of Rio de Janeiro, has removed to 43, Queen Anne Street, Cavendish Square, London, England.

